# What does the marriage of Open Access with online publication bring?

**DOI:** 10.1186/1742-6405-1-1

**Published:** 2004-12-14

**Authors:** Kailash C Gupta

**Affiliations:** 1 Division of AIDS, NIAID, National Institutes of Health, 6700B Rockledge Drive, Bethesda, MD, 0841-7626 USA

**Keywords:** Open Access, Online Publication, Journal, AIDS, HIV-1, Basic and Clinical Research, Treatment Strategies

## Abstract

Open Access online publishing is the trend of the future for unrestricted rapid and international dissemination of knowledge. Several journals are published on acquired immune deficiency syndrome (AIDS) research, but none of them appear to be Open Access. To eliminate or to abate the scourge of AIDS, it is important that the knowledge acquired through research be disseminated as soon as possible. The Open Access journal, *AIDS Research and Therapy*, is intended to fill this knowledge gap by online publication of basic, preclinical, and clinical research articles.

" The open society, the unrestricted access to knowledge, the unplanned and uninhibited association of men for its furtherance – these are what may make a vast, complex, ever growing, ever changing, ever more specialized and expert technological world, nevertheless a world of human community."

Julius Robert Oppenheimer, *Science and the Common Understanding*, Simon & Schuster, 1954 [1]

Open Access, online publication of scientific journals is a growing trend. In essence, Open Access is free and unrestricted availability of literature. This allows authors control over the integrity of their work and the right to be properly acknowledged and cited [2]. The Directory of Open Access Journals (DOAJ)  lists 1358 journals, with in excess of 61129 articles; these numbers are increasing on a daily basis. BioMed Central and the Public Library of Science (PLoS) have literally revolutionized the business of science publication and popularization. The Open Access, online publications were initially spearheaded by BioMed Central. It is interesting to reflect that the concept of Open Access scientific publications was met with disbelief and opposition by both the scientific community and the publishing houses [3], however the success of BioMed Central has shown that increasing numbers of scientific publications will occur in the Open Access area. Indeed, most scientists would enjoy disseminating their science and ideas without being encumbered by the copyright laws and restrictions imposed by traditional publishing houses. Scientists are finding that Open Access is the fastest way of communicating their research with the rest of the world without any hindrance.

AIDS is one of the biggest threats to humankind. At the end of 2003 there were 40 million HIV-infected patients worldwide, of which 3 million were estimated to have died in 2003 [4]. The number of deaths is expected to rise year after year if the spread of disease is unchecked, with an estimated 5 million new cases being added every year. In countries where the HIV infection rates are as high as 30%, it is not a matter of mere human survival, but the survival of the countries. Humankind needs to make every possible effort to rid of this scourge. It is being approached from two traditional means: prevention and treatment. We need to greatly enhance our efforts in both these approaches. It has been predicted by several mathematical models that even modest prevention efforts in the spread of HIV-1 can tame the scourge considerably.

Dissemination of the latest knowledge in basic science and its application to AIDS therapy and prevention is of paramount importance. To date, this has been accomplished with several excellent print journals dedicated to HIV-1 and the disease caused by it, AIDS. Many of them are now accessible online. However, we are launching the first Open Access journal in the AIDS arena – ***AIDS Research and Therapy***. The purpose of this journal is not to compete with or replace any of them, but to provide a unique platform where both basic research and clinical science can be studied side by side to contemplate, design, and develop applications of basic science in preclinical or clinical research. The overarching goal of the journal is to communicate emerging knowledge at a faster pace with an aim to accelerate bed-side research by taking into consideration the latest bench-side accomplishments. An additional benefit of an Open Access, online journal is enormous cost savings for libraries and institutions in terms of the high subscription rates and archival of the old journals.

*AIDS Research and Therapy *will encompass all aspects of basic and clinical science that impact on abating the spread of AIDS; it is a multidisciplinary journal that aims to keep scientists and clinicians abreast of the latest research on HIV-1. *AIDS Research and Therapy *will be particularly interested in publishing articles on novel and developing treatment strategies for AIDS, as well as articles on the outcome of treatment strategies. The ultimate goal of the journal is to lead research from bench to bedside in the combat of AIDS.

Each manuscript submitted to the journal will be sent to the appropriate Section Editor, who will assign at least 3 reviewers (members of the Editorial Board or outside experts). Reviewers are expected to return reports within two-three weeks, and the Section Editor will then make a decision based on these reports. This is to insure timely publication of findings that are important in the prevention and cure of AIDS. Indeed, we anticipate a turn around time (from submission to acceptance) of about two months. Authors, together with the Editor/reviewer handling the manuscript will be required to declare any competing interests. *AIDS Research and Therapy *will consider following types of articles: Research, Hypotheses, Methodology, Short reports, Study protocols, short as well as comprehensive Reviews. Efforts have been made to select Editors from as diverse areas of HIV and AIDS research as possible. However, Editors from areas currently not represented in the Editorial Board will be included. Readers have the option of recommending scientists who may be suitable Editors and/or reviewers.

*AIDS Research and Therapy *is published by BioMed Central, an independent publishing house, committed to ensuring peer-reviewed biomedical research is Open Access – immediately and permanently available online without charge or any other barriers to access. In an Open Access journal, the communication has to be bi-directional for the journal's success and improving the quality of the journal. Consequently, the Editorial Board of the journal will constantly seek new ideas to improve the journal to serve as an excellent resource in AIDS research and therapy to the scientific community.

Rapid dissemination of science is crucial for the progress and survival of human kind. Let the wheel of communication roll at the fastest pace and to the widest possible world.
